# The Hospitals' Celebration of the Queen's Jubilee

**Published:** 1887-01-08

**Authors:** 


### THE HOSPITALS' CELEBRATION OF THE QUEEN'S JUBILEE.

Alderman Spark, of Leeds, has shown in another column that our suggestions to the Provincial Hospitals have borne good fruit. Will not the Metropolitan Hospitals also combine and make an united effort also?

The following letter appeared in *The Times* of the 4th instant. There can be no question that if the Hospitals Association, or, as we are disposed to think, the Hospitals themselves, or, failing them, the Council of the Hospital Sunday Fund, were to take this matter up, and to set to work with a will, the £1,000,000 required might be raised within the year 1887. If this view be correct, then the financial difficulties of the Metropolitan Hospitals, and very many objectionable features in their management, would disappear once and for all. Besides, the undue and unrestrained multiplication of hospitals would be effectually prevented by this combined effort, which would render it impossible for the promoters of adventitious medical charities to secure support unless they could show cause why they have a right to demand contributions from the public :—

HOSPITAL REQUIREMENTS.

"Sir Rutherford Alcock, in his interesting letter, published in *The Times* on December 28th, summed up the hospital requirements of London, and drew conclusions which are scarcely warranted by the actual facts of the present situation. He declares that there is an ascertained and increasing deficiency in the funds provided for the maintenance of the metropolitan general hospitals and the medical charities, and that the consequent evils and deficiency of income are growing.

On page 113 of *The Hospital*, published on December 11th, it is proved by the production of actual figures that there is no falling off to-day in the charitable revenues of the 73 London hospitals which are worthy of the name, as compared with ten years ago. In brief, whereas the amount of relief given by the hospitals has increased at the rate of $\frac{1}{4}$*d.* per head of the population during the ten years in question, the voluntary income has increased to the extent of 1*d.* per head, showing a clear additional income from voluntary sources of $\frac{3}{4}$*d.* per head, or £20,000 per annum, in the voluntary income in 1885, compared with that given in 1876, when the Hospital Sunday Fund was first introduced into the metropolis. It is further shown that the undoubted monetary depression which prevails at the present time is due to the continuation of a state of affairs which existed to an equal, and possibly to a greater extent in 1876 than it does now. This monetary pressure is caused by a deficiency in income compared with the needful expenditure, of between £50,000 and £80,000 per annum. An analysis of the sources of income proves that the London hospitals have suffered much of late years, and especially in 1885, from the enormous falling off in the receipts from legacies. Thus, the London hospitals received £64,286 less from legacies in 1885 than they did in the year 1876—a falling off of more than 100 per cent.

Evidence is produced in *The Hospital* of December 18, which points to the conclusion that money formerly given in legacies to the hospitals has been diverted to other charities; seeing that seventeen miscellaneous institutions of various kinds show an increase in their revenue from legacies to an extent approaching the deficiency suffered by the hospitals in identical years. I cannot agree with Sir Rutherford Alcock that a Royal Commission at this juncture would be helpful or calculated to provide the necessary revenue. The fact that a Royal Commission was refused by three different Governments in three consecutive years, convinced the managers of the various hospitals that they must find a remedy for the existing financial difficulties by co-operation and conference with each other. As one result of this, the Hospitals Association has been founded, and now includes on its roll of members representatives of very many hospitals, asylums, nursing institutions, and workhouses throughout the country.

This spirit of co-operation spread last year to the Council of the Hospital Sunday Fund, and secured the largest contribution to the hospitals through that source which has yet been received. All these facts tend to prove that if the hospital managers will co-operate, as *The Times* recommended them to do in a leading article published on December 20, in one united effort to raise a sufficient sum—say £1,000,000, which would produce £40,000 a year—in celebration of Her Majesty's Jubilee, the financial crisis in the hospital world will be solved once and for all. Of this there can be no doubt, because the figures I have referred to prove beyond question that the present inhabitants of London subscribe more largely to the hospitals than their forefathers; and so, if the burden of debt inherited by the hospital managers can be removed by a Jubilee Fund once and for all, neither a Royal Commission nor any other action will be necessary on the part of the Government.

Cannot some steps be taken, therefore, to call a meeting of metropolitan hospital managers to discuss the feasibility of celebrating Her Majesty's Jubilee by placing the London hospitals in a permanently sound financial position? I am, Sir, your obedient servant,

The Editor of *The Hospital*."

Mr. Charles Hawkins, who has taken a deep interest in St. George's Hospital for very many years, sends us a statement giving full particulars of the income and expenditure of that institution, from its foundation in 1733. It appears that the *total receipts*, excluding stock purchased or acquired by wills—an important item, by the way, which was increased by £90,000 in one sum in 1885—and cash received for stock sold, amounted to £1,368,337, and *the ordinary expenditure* to £1,371,957, giving a total deficiency in income of less than £4,000 for the 153 years in question; truly a remarkable record. It will, therefore, be seen that, although St. George's Hospital sold more than a quarter-of-a-million of stock, much of this must have been replaced to capital account, seeing that *the extraordinary expenses on buildings*, etc., not included in *the ordinary expenditure* amounted to only £130,161. This is another proof that the voluntary income of the chief metropolitan hospitals has not fallen off, but increased of late years, and that the existing financial depression is due to the accumulation of old debt acquired in former times.

